# Association between non‐alcoholic fatty liver disease with the susceptibility and outcome of COVID‐19: A retrospective study

**DOI:** 10.1111/jcmm.17042

**Published:** 2021-11-10

**Authors:** Guyi Wang, Shangjie Wu, Chenfang Wu, Quan Zhang, Fang Wu, Bo Yu, Siye Zhang, Chao Wu, Guobao Wu, Yanjun Zhong

**Affiliations:** ^1^ Critical Care Medicine The Second Xiangya Hospital Central South University Changsha China; ^2^ Department of Respiratory Medicine The Second Xiangya Hospital Central South University Changsha Hunan China; ^3^ Critical Care Medicine The First Hospital of Changsha China; ^4^ Department of Oncology The Second Xiangya Hospital Central South University Changsha Hunan China; ^5^ Department of Metabolism & Endocrinology The Second Xiangya Hospital Central South University Changsha Hunan China

**Keywords:** body mass index, COVID‐19, liver injury, non‐alcoholic fatty liver disease, outcome, susceptibility

## Abstract

This study aims to evaluate the effect of non‐alcoholic fatty liver disease (NAFLD) on the susceptibility and consequences of coronavirus disease 2019 (COVID‐19). We retrospectively collected data from 218 adult COVID‐19 patients who showed no evidence of excessive alcohol consumption and underwent abdominal ultrasound examinations. Of these patients, 39.4% patients had been diagnosed with NAFLD, which indicates a much higher prevalence of NAFLD than that reported in the general population. Significantly elevated white blood cell count (*p* = 0.008), alanine aminotransferase (*p* = 0.000), aspartate aminotransferase (*p* = 0.006) and C reactive protein (*p* = 0.012) were found in the patients with NAFLD. These patients also had significantly higher proportions of hypertension (*p* = 0.006) and diabetes (*p* = 0.049) than the non‐NAFLD cases. No significant differences existed in the severity, mortality, viral shedding time and length of hospital stay between patients with or without NAFLD in the sample population. However, subgroup analyses found that in patients with normal body mass index (BMI), NAFLD sufferers were more likely to experience a severe event (30.0% vs 11.5%, *p* = 0.021). Kaplan‐Meier curve (log‐rank *p* = 0.017) and Cox regression (HR = 3.26, 95% CI: 1.17–9.04, *p* = 0.023) analyses confirmed that before and after adjusting for gender, age and comorbidities, NAFLD patients with normal BMI had a higher incidence of suffering severe events. People with NAFLD may have a higher proportion of COVID‐19. NAFLD may be correlated with the severity of COVID‐19 patients in the normal BMI group.

## 1 INTRODUCTION

Coronavirus disease 2019 (COVID‐19) was first reported in Wuhan, China, in December 2019^1^, ^2^, ^3^, ^4^, ^5^ and now has spread all around the world.^6^, ^7^ Recently, several reports have discovered that a large number of COVID‐19 patients had underlying conditions, especially in severe and lethal cases.^3^, ^4^, ^8^ Also, pre‐existing ailments may be closely related to the susceptibility and poor outcomes of COVID‐19.^9^ Liver impairment has been reported in up to 50% of patients with COVID‐19, and more than 60% of severe cases.^10^ Histological examination in the liver of a COVID‐19 patient showed moderate micro‐vesicular steatosis and mild lobular and portal activity.^11^ However, the causes of liver injury in COVID‐19 cases and the effects of liver‐related comorbidities on the susceptibility and outcomes of COVID‐19 remain unclear.

Non‐alcoholic fatty liver disease (NAFLD) is an acquired metabolic stress‐related liver disorder, with an overall global prevalence of about 25%.^12^ The incidence of NAFLD in China is rising rapidly, from about 23.8% in the early 2000s to about 32.9% in 2018.^12^, ^13^, ^14^, ^15^ NAFLD often coexists with hyperlipidaemia and/or obesity.^16^, ^17^, ^18^, ^19^ Therefore, this study aims to evaluate the relationship between NAFLD and COVID‐19 using a retrospective cohort of Chinese individuals diagnosed with COVID‐19.

## 2 METHODS (SUBJECTS) AND MATERIALS

### 2.1 Study Design and Participants

The study protocol was subject to approval by the institutional ethics board of the Second Xiangya Hospital of Central South University (No. 2020001). We retrospectively collected data from a cohort of subjects who had laboratory‐confirmed COVID‐19 and were patients in the Public Health Treatment Center of Changsha, China, before 14 March 2020. Fatty liver was diagnosed based on the ultrasound parameters (such as parenchymal brightness, deep beam attenuation and liver‐to‐kidney contrast) by abdominal ultrasonography with a 3.5‐MHz transducer (S9, SonoScape, China). Ultrasonography was performed by the experienced radiologists for medical indications in the Public Health Treatment Center of Changsha, China. We used the following criteria to diagnose NAFLD: (1) The presence of fatty liver; (2) The absence of excessive alcohol consumption (average alcohol intake ≥30 g/day for men and ≥20 g/day for women).

### 2.2 Data Collection

Two authors carefully collected and reviewed the individual medical records of the patients. Detailed information on demographic data, body mass index (BMI), underlying comorbidities, medical history, symptoms, laboratory parameters, chest computed tomographic (CT) scans and outcomes were recorded. For BMI data, the medical records of 18 patients did not include height and weight.

### 2.3 Endpoints

The primary endpoint of this study was a severe event, which was defined using the following criteria: (1) respiratory rate ≥30 breaths/min; (2) oxygen saturation <93%; (3) PaO2/FiO2 ≤ 300 mmHg; (4) lung lesions progressed to greater than 50% within 24–48 hours; (5) mechanical ventilation was implemented; (6) shock; (7) combined with other organ failures, required intensive care.^20^ Secondary endpoints included the mortality rate, virus shedding time and length of hospital stay.

### 2.4 Statistical Analysis

Because the continuous variables in this study were non‐normal distributed, we used the Fisher's exact test (χ2 test) and Mann‐Whitney test to compare the differences between the categorical variables and the continuous variables, separately. The Kaplan‐Meier (KM) method and the log‐rank test were applied to assess the association between NAFLD and the outcomes. Cox regression was conducted to further evaluate the effect of NAFLD after adjusting for other risk factors. All analyses were performed using IBM SPSS Statistics version 26.0 software.

## 3 RESULTS

**FIGURE 1:**
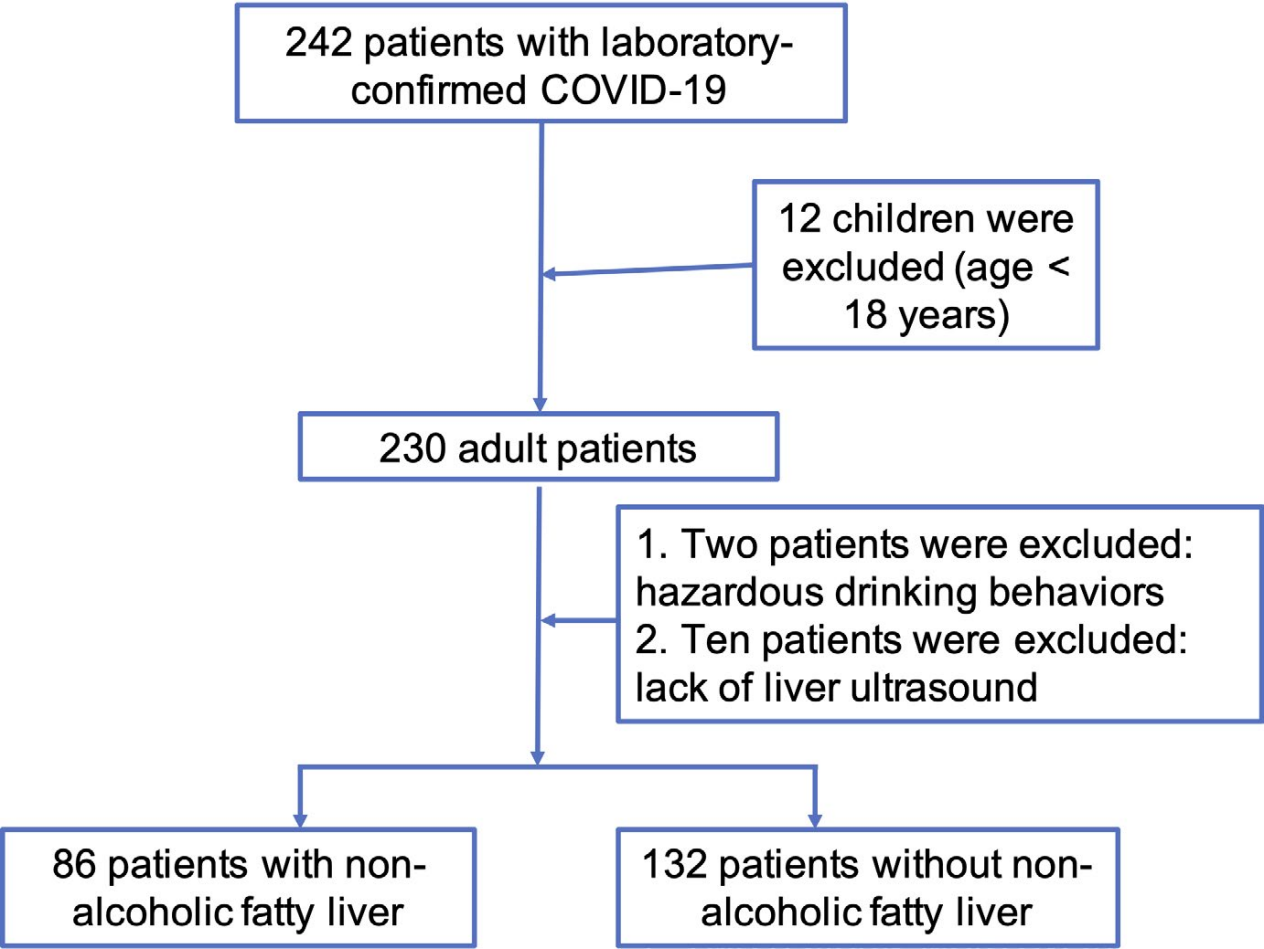
Flow chart of the study population

In this study, we retrospectively collected the data of 230 adult patients who had contracted laboratory‐confirmed COVID‐19 before 14 March 2020, in the Public Health Treatment Center of Changsha, China. Of these patients, 218 cases had undergone abdominal ultrasound examinations and did not display the presence of excessive alcohol consumption (Figure 1). A total of 86 patients (39.4%) had been diagnosed with NAFLD, which presented a higher incidence of NAFLD than what has been reported in the general population of China. Of the NAFLD patients, 52 (60.5%) were males. Compared with the non‐NAFLD group, patients with NAFLD had a higher BMI (24.8 kg/m^2^ [18.7–37.2] vs 21.8 kg/m^2^ [11.7–29.3], *p* = 0.000) and higher proportions of hypertension (23.3% vs 9.1%, *p* = 0.004) and diabetes (10.5% vs 3.8%, *p* = 0.049). However, no significant differences were found in other comorbidities (including cardiovascular disease and chronic liver disease) and common COVID‐19 symptoms (including fever, cough, fatigue and expectoration). Two of the patients (1.5%) died, while the remaining 216 patients were discharged before 14 March 2020 (Table 1). The two patients who died did not suffer from NAFLD.

**TABLE 1:** Baseline Characteristics and laboratory findings on admission of COVID‐19 patients with and without NAFLD

	NAFLD (n = 86)	Non‐NAFLD (n= 132)	p value
Sex (male/female)	52/34	58/74	0.017
Age, y, median (range)	46 (19–76)	45 (21–84)	0.895
BMI, kg/m^2^, median (range)	24.8 (18.7–37.2)	21.8 (11.7–29.3)	0.000
Hypertension (n, %)	20 (23.3%)	12 (9.1%)	0.004
CVD (n, %)	3 (3.5%)	5 (3.8%)	1.000
Diabetes (n, %)	9 (10.5%)	5 (3.8%)	0.049
Chronic liver disease (n, %)	5 (5.8%)	6 (4.5%)	0.919
Fever (n, %)	66 (76.7%)	97 (73.5%)	0.588
Cough (n, %)	75 (87.2%)	104 (78.8%)	0.113
Fatigue (n, %)	39 (45.3%)	61 (46.2%)	0.901
Expectoration (n, %)	38 (44.2%)	61 (46.2%)	0.769
WBC, x10^9^/L, median (range)	5.0 (1.9–13.4)	4.3 (0.8–10.4)	0.008
Lys count, x10^9^/L, median (range)	1.1 (0.4–3.7)	1.1 (0.1–3.1)	0.344
ALT, U/L, median (range)	26.2 (11.3–93.7)	16.8 (2.6–69.4)	0.000
AST, U/L, median (range)	25.7 (2.0–78.8)	23.3 (12.3–82.1)	0.006
Total bilirubin, μmol/L, medium (range)	11.8 (4.3–38.2)	10.3 (4.0–162.1)	0.105
Creatinine, μmol/L, medium (range)	53.7 (28.6–105.1)	50.5 (20.6–255.7)	0.256
Creatine kinase, U/L, medium (range)	85.5 (11.3–986.4)	66.5 (22.7–646.0)	0.002
Creatine kinase‐MB, U/L, medium (range)	9.4 (0.4–82.8)	9.6 (0.3–221.7)	0.953
Total cholesterol, mg/dl, medium (range)	3.9 (2.3–6.9)	3.6 (1.9–6.4)	0.018
LDL cholesterol, mg/dl, medium (range)	2.7 (1.5–5.9)	2.5 (1.4–4.9)	0.006
HDL cholesterol, mg/dl, medium (range)	0.7 (0.5–1.2)	0.8 (0.2–1.6)	0.000
Triglyceride, mg/dl, medium (range)	1.4 (0.9–6.4)	0.9 (0.1–2.7)	0.000
CRP, mg/L, median (range)	21.3 (0.4–94.9)	13.1 (0.1–101.9)	0.012
PCT, ≥0.05 ng/mL (n, %)	31 (36.0%)	29 (22.0%)	0.023
Chest CT positive rate (n, %)	82 (95.3%)	125 (94.7%)	0.971
Chest CT with ground‐glass change	50 (58.1%)	50 (37.9%)	0.003
Severe events (n, %)	19 (22.1%)	22 (16.7%)	0.316
Virus shedding time (days)	17 (3–47)	18 (6–53)	0.165
Length of hospital stay (days)	15 (5–41)	16 (5–40)	0.407
Mortality (n, %)	0 (0%)	2 (1.5%)	0.251

Abbreviations: NAFLD, non‐alcoholic fatty liver disease; y, years; CVD, cardiovascular disease; WBC, white blood cell count; Lys, lymphocyte; ALT, alanine aminotransferase; AST, aspartate aminotransferase; CRP, C reactive protein; PCT, procalcitonin.

In terms of laboratory findings, considerably higher levels of white blood cells (5.0 x 10^9^/L [1.9–13.4] vs 4.3 x 10^9^/L [0.8–10.4]; *p* = 0.008), alanine aminotransferase (26.2 U/L [11.3–93.7] vs 16.8 U/L [2.6–69.4]; *p* = 0.000), aspartate aminotransferase (25.7 U/L [2.0–78.8] vs 23.3 U/L [12.3–82.1]; *p* = 0.006), creatine kinase (85.5 U/L [11.3–986.4] vs 66.5 U/L [22.7–646.0], *p* = 0.002) and C reactive protein (21.3 mg/L [0.4–94.9] vs 13.1 mg/L [0.1–101.9]; *p* = 0.012) were detected in the patients with NAFLD than in those without NAFLD. Meanwhile, the proportions of procalcitonin positive subjects (36.0% vs 22.0%, *p* = 0.023) and the incidence of ground‐glass opacity obtained from chest CTs (58.1% vs 37.9%, *p* = 0.003) were significantly higher in the NAFLD group than in the non‐NAFLD group (Table 1). This may suggest that patients with NAFLD had a higher inflammatory response to SARS‐CoV‐2 infection and a higher incidence of liver injury. Moreover, significantly differences were observed in the serum levels of lipid metabolism, including total cholesterol (3.9 mg/dL [2.3–6.9] vs 3.6 mg/dL [1.9–6.4], *p* = 0.018), low‐density lipoprotein (LDL) cholesterol (2.7 mg/dL [1.5–5.9] vs 2.5 mg/dL [1.4–4.9], *p* = 0.006), high‐density lipoprotein (HDL) cholesterol (0.7 mg/dL [0.5–1.2] vs 0.8 mg/dL [0.2–1.6], *p* = 0.000) and triglyceride (1.4 mg/dL [0.9–6.4] vs 0.9 mg/dL [0.1–2.7], *p* = 0.000; Table 1).

With regard to the outcomes, there was no significant difference between NAFLD and non‐NAFLD patients, including the severe events (22.1% vs 16.7%, *p* = 0.316), virus shedding time (17 days [3–47] vs 18 days [6–53], *p* = 0.165), length of hospital stay (15 days [5–41] vs 16 days [5–40], *p* = 0.407) and mortality (0% vs 1.5%, *p* = 0.251).

**FIGURE 2:**
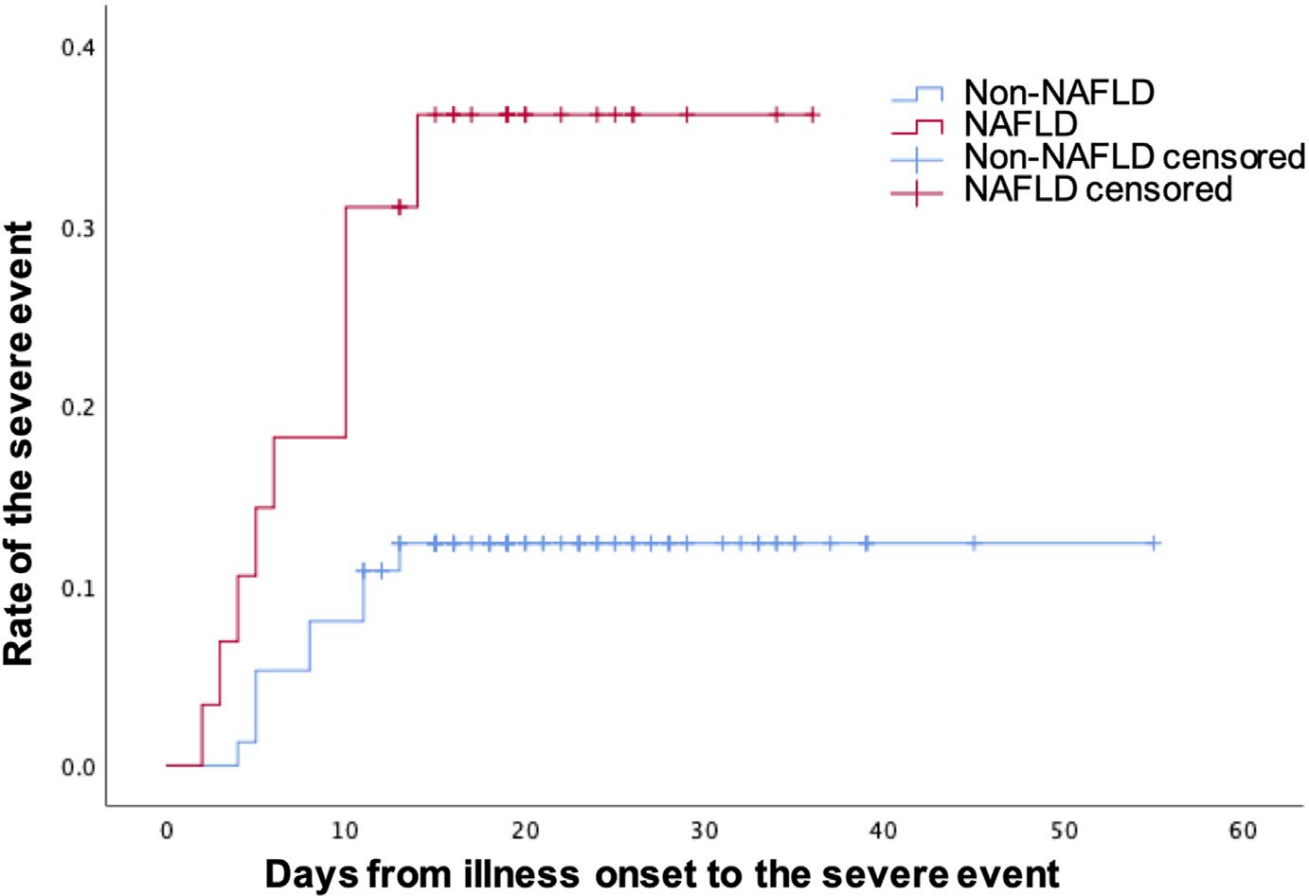
Time‐dependent risk of suffering a severe event between COVID‐19 patients of normal BMI with non‐alcoholic fatty liver disease (NAFLD) and without NAFLD

Next, to investigate whether the effect of NAFLD on the outcomes of COVID‐19 patients was dependent on gender, age, BMI or comorbidities, we divided our patients into subgroups according to these factors. We found that in patients with normal BMI (18.5 ≤ BMI <24 kg/m^2^), NAFLD patients were more likely to develop severe events than those without NAFLD (30.0% vs 11.5%, *p* = 0.021). However, no significant differences were found in patients with low (< 18.5 kg/m^2^) or high (≥ 24 kg/m^2^) BMI values (Table 2). Regarding laboratory parameters, NAFLD patients in the normal BMI group had significantly higher levels of alanine aminotransferase (26.2 U/L [12.1–93.7] vs 15.8 U/L [4.9–58.4], *p* = 0.000), aspartate aminotransferase (25.7 U/L [11.5–58.5] vs 22.6 U/L [12.6–69.0], *p* = 0.009), total bilirubin (12.6 μmol/L [6.9–30.1] vs 9.7 μmol/L [4.0–26.1], *p* = 0.010), C reactive protein (20.8 mg/L [1.0–94.9] vs 11.1 mg/L [0.1–101.9], *p* = 0.012) and triglyceride (1.4 mg/dL [0.9–6.1] vs 0.9 mg/dL [0.1–2.7], *p* = 0.000). Conversely, they had slightly lower HDL cholesterol levels (0.8 mg/dL [0.6–1.2] vs 0.9 mg/dL [0.5–1.6], *p* = 0.008) than those without NAFLD (Table 3). Thus, a KM analysis was performed and confirmed that NAFLD patients were more likely to experience a severe event (log‐rank *p* = 0.017; Figure 2). After adjusting for gender, age, hypertension, cardiovascular disease, diabetes and chronic liver disease, NAFLD is significantly correlated with the severity of COVID‐19 (HR =3.26, 95% CI: 1.17–9.04, *p* = 0.023; Table 4) in the normal BMI group.

**TABLE 2:** Influence of NAFLD on the severe event in subgroups of COVID‐19 patients according to gender, age, BMI and comorbidities

	Severe event (n, %)		p value	Virus shedding time (days)		p value	Length of hospital stay (days)		p value
	NAFLD (n=86)	Non‐NAFLD (n = 132)		NAFLD (n=86)	Non‐NAFLD (n = 130)		NAFLD (n=86)	Non‐NAFLD (n = 130)	
Gender									
Male	12 (23.1%)	13 (22.4%)	0.934	17 (6–47)	17.5 (6–42)	0.696	14 (5–41)	17 (5–37)	0.689
Female	7 (20.6%)	9 (12.2%)	0.252	17.5 (1–38)	19 (5–53)	0.131	17 (5–37)	16 (5–40)	0.569
Age									
Elderly (≥ 60 years)	5 (25%)	13 (35.1%)	0.432	16 (4–38)	23 (11–43)	0.011	17 (6–37)	21 (6–40)	0.155
Non‐elderly (< 60years)	14 (21.2%)	9 (10.5%)	0.036	17.5 (3–47)	17 (6–53)	0.975	15 (5–41)	15 (5–40)	0.986
BMI									
BMI ≥24 kg/m^2^	7 (14.6%)	7 (23.3%)	0.327	18.5 (8–47)	21 (6–45)	0.595	15 (5–41)	17 (6–40)	0.643
18.5 ≤ BMI <24 kg/m^2^	9 (30%)	9 (11.5%)	0.021	17 (3–38)	17 (8–53)	0.939	16.5 (5–37)	16 (5–37)	0.697
BMI <18.5 kg/m^2^	NA	3 (21.4%)	NA	NA	18.5 (8–40)	NA	NA	14.5 (8–40)	NA
Hypertension									
Yes	9 (45%)	4 (33.3%)	0.780	16.5 (11–45)	18 (12–43)	0.366	16.5 (5–41)	21 (8–35)	0.387
No	10 (15.2%)	18 (15.0%)	0.978	17.5 (3–47)	18.5 (6–53)	0.138	16.5 (11–45)	18 (12–43)	0.348
Cardiovascular disease									
Yes	2 (66.7%)	2 (40.0%)	1.000	21 (19–29)	35 (22–29)	0.114	22 (19–27)	24.5 (14–35)	0.857
No	17 (20.5%)	20 (15.7%)	0.379	17 (3–47)	18 (6–53)	0.205	15 (5–41)	16 (5–40)	0.411
Diabetes									
Yes	2 (22.2%)	2 (40.0%)	0.580	15 (9–29)	27 (15–43)	0.112	15 (6–30)	28 (17–32)	0.060
No	17 (22.1%)	20 (15.7)	0.255	17 (3–47)	18 (6–53)	0.259	15 (5–41)	16 (5–40)	0.560
Chronic liver disease									
Yes	2 (40.0%)	1 (16.7%)	0.545	16 (14–29)	24 (11–34)	0.537	13 (9–27)	22 (8–36)	0.662
No	17 (21.0%)	21 (16.7%)	0.433	17 (3–47)	18 (6–53)	0.188	15 (5–41)	15 (5–40)	0.496

Abbreviations: NAFLD, Non‐alcohol fatty liver disease; BMI, Body mass index; NA, Not available.

**TABLE 3:** Baseline Characteristics and laboratory findings on admission of COVID‐19 patients of normal BMI with and without NAFLD

	NAFLD (n = 30)	Non‐NAFLD (n=78)	p value
Sex (male/female)	15/15	45/33	0.471
Age, y, median (range)	47 (27–72)	43 (21–84)	0.929
Hypertension (n, %)	8 (26.7%)	9 (11.5%)	0.101
CVD (n, %)	2 (6.7%)	3 (3.8%)	0.616
Diabetes (n, %)	3 (10.0%)	4 (5.1%)	0.628
Chronic liver disease (n, %)	3 (10.0%)	4 (5.1%)	0.628
Fever (n, %)	25 (83.3%)	56 (71.8%)	0.173
Cough (n, %)	26 (86.7%)	55 (70.5%)	0.082
Fatigue (n, %)	18 (60.0%)	34 (43.6%)	0.126
Expectoration (n, %)	16 (53.3%)	27 (34.6%)	0.075
WBC, x10^9^/L, median (range)	4.6 (2.3–9.35)	4.5 (0.8–10.4)	0.384
Lys count, x10^9^/L, median (range)	1.0 (0.4–2.7)	1.2 (0.1–3.1)	0.131
ALT, U/L, median (range)	26.2 (12.1–93.7)	15.8 (4.9–58.4)	0.000
AST, U/L, median (range)	25.7 (11.5–58.5)	22.6 (12.6–69.0)	0.009
Total bilirubin, μmol/L, medium (range)	12.6 (6.9–30.1)	9.7 (4.0–26.1)	0.010
Creatinine, μmol/L, medium (range)	52.0 (30.0–83.3)	50.9 (20.6–213.9)	0.970
Creatine kinase, U/L, medium (range)	104.7 (17.4–513.3)	68.4 (22.7–449.5)	0.054
Creatine kinase‐MB, U/L, medium (range)	11.2 (1.1–34.1)	9.7 (0.3–35.2)	0.259
CRP, mg/L, median (range)	20.8 (1.0–94.9)	11.1 (0.1–101.9)	0.012
Total cholesterol, mg/dL, medium (range)	3.9 (2.5–6.9)	3.8 (2.5–5.9)	0.380
LDL cholesterol, mg/dL, medium (range)	2.7 (1.6–5.9)	2.6 (1.4–4.4)	0.344
HDL cholesterol, mg/dL, medium (range)	0.8 (0.6–1.2)	0.9 (0.5–1.6)	0.008
Triglyceride, mg/dL, medium (range)	1.4 (0.9–6.1)	0.9 (0.1–2.7)	0.000
PCT, ≥0.05 ng/mL (n, %)	9 (30.0%)	16 (20.5%)	0.295
Chest CT positive rate (n, %)	29 (96.7%)	74 (94.9%)	1.000
Chest CT with ground‐glass change	18 (60%)	31 (39.7%)	0.058
Virus shedding time (days)	17 (3–38)	17 (6–53)	0.989
Length of hospital stay (days)	16.5 (5–37)	16 (5–37)	0.695

Abbreviations: NAFLD, non‐alcoholic fatty liver disease; y, years; CVD, cardiovascular disease; WBC, white blood cell count; Lys, lymphocyte; ALT, alanine aminotransferase; AST, aspartate aminotransferase; CRP, C reactive protein; PCT, procalcitonin.

**TABLE 4:** Multivariate analysis of factors related to severe events of COVID‐19 patients with normal body mass index using the COX regression model

	HR (hazard ratio)	95% confidence interval	p value
NAFLD	3.26	1.17, 9.04	0.023
Gender	1.91	0.71, 5.20	0.203
Age	1.04	1.00, 1.08	0.049
Hypertension	1.12	0.29, 4.32	0.873
Cardiovascular disease	0.36	0.07, 1.85	0.220
Diabetes	0.72	0.15, 3.44	0.678
Chronic liver disease	0.90	0.14, 5.69	0.913

Abbreviation: NAFLD, non‐alcoholic fatty liver disease.

Moreover, the results showed that in non‐elderly patients (< 60 years), those with NAFLD had a higher incidence of severe events (21.2% vs 10.5%, *p* = 0.036; Table 2). Subsequently, a KM analysis confirmed that non‐elderly NAFLD patients had a significantly elevated risk of suffering a severe event compared with those without NAFLD (log‐rank *p* = 0.035; Figure S1). However, after adjusting for gender, hypertension, cardiovascular disease, diabetes and chronic liver disease, NAFLD had no significant influence on the likelihood of the severe events in non‐elderly patients (HR =1.79, 95% CI: 0.72–4.44, *p* = 0.208; Table S2).

No significant effects of NAFLD were found on the virus shedding time or length of hospital stay in the patient cohort. Thus, we performed subgroup analyses according to gender, age, BMI and the common comorbidities. Results showed that in elderly patients, the virus shedding time was significantly lower in NAFLD patients than in non‐NAFLD individuals (16 days [4–38] vs 23 days [11–43], *p* = 0.011; Table 2). In terms of laboratory findings among elderly patients, alanine aminotransferase was conspicuously higher in patients with NAFLD, compared to those without (25.0 U/L [12.8–58.1] vs 15.6 U/L [4.9–69.4], *p* = 0.002; Table S1).

## 4 DISCUSSION

This study investigated the susceptibility of patients with NAFLD to SARS‐CoV‐2 and the association between NAFLD and outcomes of COVID‐19. We found that individuals with NAFLD may be more susceptible to SARS‐CoV‐2 than those without NAFLD. In patients with normal BMI, those with NAFLD who were infected by COVID‐19 may have a higher proportion of developing severe diseases than other patients.

In our study, 39.4% of the COVID‐19 patients we investigated had NAFLD, which is significantly higher than the rate reported in the general population.^12^, ^13^, ^14^, ^15^ This finding is consistent with another study that was done in China.^21^ It suggests that NAFLD sufferers generally seem to be more susceptible to SARS‐CoV‐2 infection than people without NAFLD. Although the reason for this is not yet known, immune system disorders in NALFD may be an important cause, such as a decrease in CD4 ^+^ T cells and abnormal macrophage function.^22^, ^23^

NAFLD often coexists with hyperlipidaemia and obesity.^17^, ^18^ People with NAFLD usually have a high incidence of hypertension, diabetes and cardiovascular disease. All of these conditions have been demonstrated to be independent risk factors of COVID‐19.^24^ In this study, compared with the non‐NAFLD patients, the proportions of hypertension and diabetes were also significantly higher in the NAFLD group, which is similar to the conclusions of previous studies.

Despite no evidence of increased liver uptake of SARS‐CoV‐2 in NAFLD patients,^25^ in our study, the COVID‐19 cases with NAFLD had higher levels of alanine aminotransferase and aspartate aminotransferase than the other patients. Moreover, patients with abnormal liver tests are at a higher risk of developing to the severe events.^26^ It has been reported that patients with NAFLD have a higher risk of disease progression,^21^ while NAFLD patients with an increased fibrosis‐4 index and NAFLD fibrosis score are at a higher risk of suffering a severe event due to COVID‐19.^27^ However, the influence of NAFLD on the COVID‐19 in subgroups of age, gender, BMI and comorbidities remains unclear.

The prevalence and severity of NAFLD vary between different age groups.^15^, ^28^ A large sample study in China found that, compared to non‐elderly adults (18–59 years), the elderly (≥ 60 years) showed a lower overall prevalence of NAFLD, but a higher proportion of NAFLD accompanied with diabetes, hypertension and hyperlipidaemia.^29^ Elderly NAFLD patients are more likely to develop non‐alcoholic steatohepatitis and fibrosis than non‐elderly patients.^30^ Moreover, NAFLD is considered to be associated with increased mortality in elderly people.^31^ Interestingly, in the non‐elderly group of our study, NAFLD is significantly associated with the severity of COVID‐19. After adjusting for gender, hypertension and BMI, the difference in frequency of the severe events becomes statistically insignificant, which may be a result of both the high incidence of comorbidities and the small sample size.

The susceptibility to COVID‐19 of NAFLD sufferers increases linearly with BMI. It increases 5‐ to 10‐fold in obese patients and by a factor of 10‐ to 14‐fold in the morbidly obese.^32^ However, a significant proportion of NAFLD patients have relatively normal BMI, and it has been reported that obese NAFLD has different clinical characteristics from non‐obese NAFLD.^33^ Therefore, in this study, we performed a subgroup analysis on BMI and found that in patients with a normal BMI (18.5–24 kg/m^2^), NAFLD was associated with an increased risk of severe disease. However, we did not find similar results in patients with higher and lower BMI, which may be related to their different metabolic states. This is an interesting result, but well‐designed studies with larger sample sizes are required to investigate this assumption.

This study contains several limitations. Firstly, we analysed the effect of NAFLD on the susceptibility and outcome of COVID‐19 in one of the nearest provincial capitals to Wuhan, but we did not have the data on the prevalence of NAFLD in Changsha, China. Also, in most of the subgroups that were created according to the common comorbidities, no significant differences were found. However, the results did show certain trends. Univariate analysis indicated that non‐elderly NAFLD patients had a higher incidence of severe events. Nevertheless, after adjusting for gender, hypertension and BMI, the difference became statistically insignificant. This may be because of the small sample size and the high incidence of comorbidities. Another limitation is that in the subgroup analysis on BMI, we only divided our patients into three groups. Overweight and obese patients were pooled together in one group (BMI ≥24 kg/m^2^), due to the small number of obese patients (BMI ≥28 kg/m2). Finally, as this study was retrospective, data regarding abdominal adiposity was not available. Therefore, well‐designed studies with larger sample sizes are still needed to more clearly demonstrate the association between NAFLD and the outcomes of COVID‐19.

In summary, people with NAFLD may have a higher proportion of SARS‐CoV‐2 infection, with a higher rate of liver injury, as well as a higher incidence of severe COVID‐19 in normal‐weight patients. People who have NAFLD may need stronger protection and more aggressive treatment to decrease the risk of infection, reduce the chance of liver injury and improve the outcome. However, due to the small sample size, especially for elderly patients, further studies with a larger sample size are required to confirm these findings.

## CONFLICT OF INTEREST

The authors confirm that there are no conflicts of interest.

## AUTHOR CONTRIBUTION

**Guyi Wang:** Conceptualization (equal); Data curation (equal); Formal analysis (equal); Investigation (equal); Methodology (equal); Project administration (equal); Resources (equal); Writing‐original draft (equal); Writing‐review & editing (equal). **Shangjie Wu:** Funding acquisition (equal); Investigation (equal); Supervision (equal); Validation (equal); Writing‐review & editing (equal). **Chenfang Wu:** Data curation (equal); Investigation (equal); Supervision (equal); Writing‐review & editing (equal). **Quan Zhang:** Data curation (equal); Formal analysis (equal); Investigation (equal); Methodology (equal); Resources (equal). **Fang Wu:** Formal analysis (equal); Investigation (equal); Supervision (equal); Writing‐review & editing (equal). **Bo Yu:** Conceptualization (equal); Data curation (equal); Methodology (equal). **Siye Zhang:** Data curation (equal); Formal analysis (equal); Methodology (equal). **Chao Wu:** Data curation (equal); Formal analysis (equal); Writing‐original draft (equal). **Guobao Wu:** Supervision (equal); Writing‐review & editing (equal). **Yanjun Zhong:** Conceptualization (equal); Data curation (equal); Formal analysis (equal); Funding acquisition (equal); Investigation (equal); Methodology (equal); Project administration (equal); Resources (equal); Supervision (equal); Validation (equal); Visualization (equal); Writing‐original draft (equal); Writing‐review & editing (equal).

## Supporting information

Fig S1

Table S1

Table S2
