# Supplementary figures and images for: Association between non‐alcoholic fatty liver disease with the susceptibility and outcome of COVID‐19: A retrospective study
Source: J Cell Mol Med. 2021 Nov 10;25(24):11212–20. doi: 10.1111/jcmm.17042 (PMC8650045; doi:10.1111/jcmm.17042)

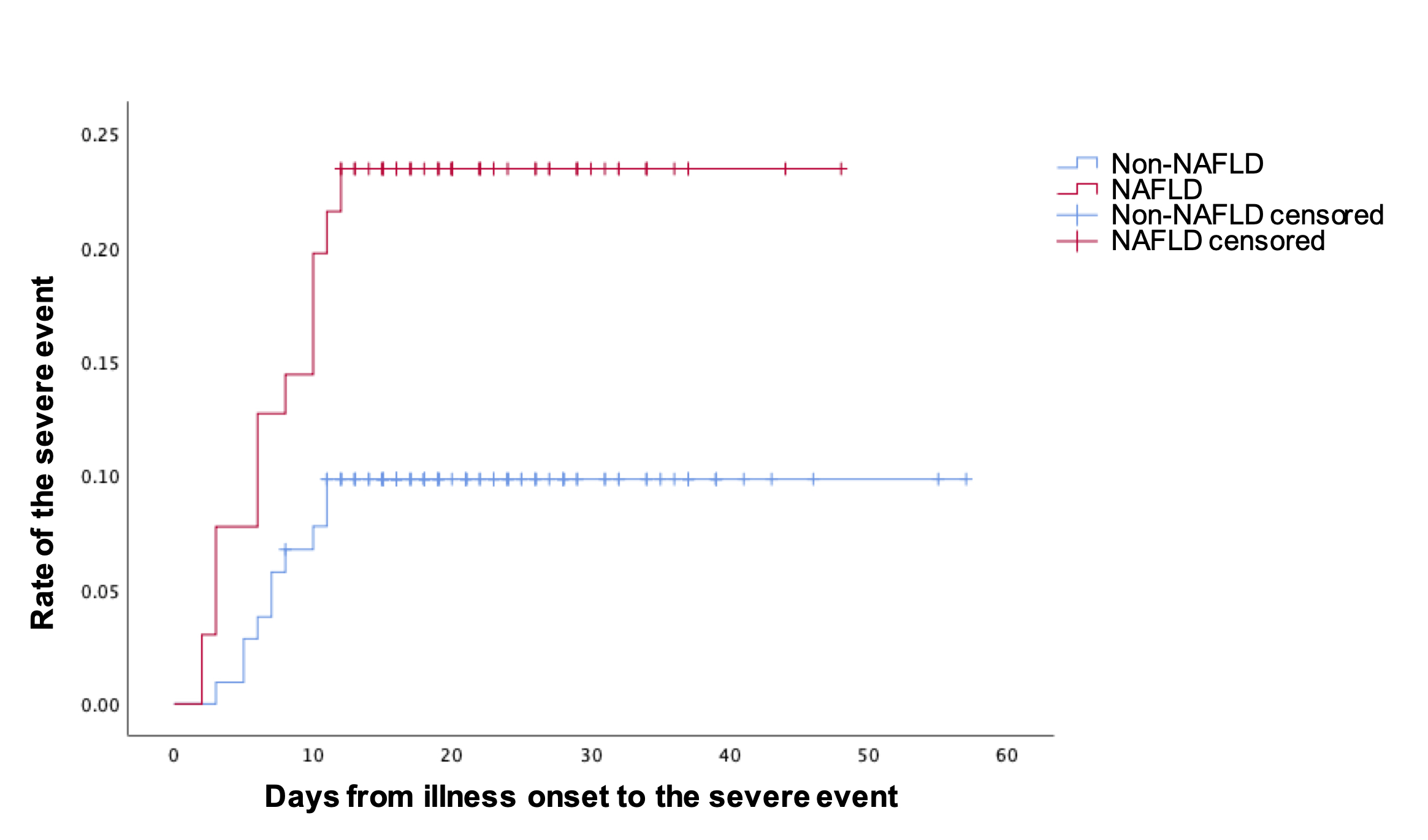

Supplement: Supplementary file 1 — Fig S1 [file JCMM-25-11212-s003.jpg]
