# Supplementary material for: Association between non‐alcoholic fatty liver disease with the susceptibility and outcome of COVID‐19: A retrospective study
Source: J Cell Mol Med. 2021 Nov 10;25(24):11212–20. doi: 10.1111/jcmm.17042 (PMC8650045; doi:10.1111/jcmm.17042)
Supplement: Supplementary file 2 — Table S1 [file JCMM-25-11212-s001.docx]

**Table S1 Baseline Characteristics and laboratory findings** **on admission of elderly and non-elderly COVID-19 patients with and without NAFLD**

|  | Elderly (≥ 60 y) | | | Non-elderly (18-59 y) | | |
| --- | --- | --- | --- | --- | --- | --- |
|  | **NAFLD**  **(n = 20)** | **Non-NAFLD**  **(n = 37)** | **p value** | **NAFLD**  **(n= 66)** | **Non-NAFLD**  **(n= 95)** | **p value** |
| Sex (male/female) | 7/13 | 15/22 | 0.682 | 45/21 | 43/52 | **0.004** |
| Age, y, M (range) | 66 (60-76) | 67 (60-84) | 0.355 | 41 (19-59) | 40 (21-59) | 0.360 |
| Hypertension (n, %) | 11 (55.0%) | 9 (24.3%) | **0.021** | 9 (13.6%) | 3 (3.2%) | **0.029** |
| CVD (n, %) | 2 (10.0%) | 4 (10.8%) | 1.000 | 1 (1.5%) | 1 (1.1%) | 1.000 |
| Diabetes (n, %) | 6 (30.0%) | 4 (10.8%) | 0.146 | 3 (4.5%) | 1 (1.1%) | 0.306 |
| WBC, x10^9^/L, M (range) | 4.8 (2.3-6.5) | 4.8 (0.8-9.6) | 0.770 | 5.2 (1.9-13.4) | 4.1 (2.3-10.4) | **0.004** |
| Lys count, x10^9^/L, M (range) | 1.0 (0.4-1.9) | 0.9 (0.1-2.2) | 0.604 | 1.2 (0.5-3.7) | 1.2 (0.4-3.1) | 0.520 |
| ALT, U/L, M (range) | 25.0 (12.8-58.1) | 15.6 (4.9-69.4) | **0.002** | 26.7 (11.3-93.7) | 16.8 (2.6-58.4) | **0.000** |
| AST, U/L, M (range) | 33.1 (17.8-59.5) | 26.9 (12.6-80.5) | 0.249 | 24.6 (2.0-78.8) | 21.8 (12.3-82.1) | **0.004** |
| CRP, mg/L, M (range) | 39.7 (0.4-91.4) | 21.4 (0.2-101.9) | 0.085 | 13.1 (0.7-94.9) | 11.4 (0.1-76.6) | **0.042** |
| PCT, ≥0.05 nmol/L (n, %) | 11 (55.0%) | 10 (27.0%) | **0.037** | 20 (30.3%) | 19 (20.0%) | 0.133 |

Notes: NAFLD, non-alcoholic fatty liver disease; M, median; y, years; CVD, cardiovascular disease; WBC, white blood cell count; Lys, lymphocyte; ALT, alanine aminotransferase; AST, aspartate aminotransferase; CRP, C reactive protein; PCT, procalcitonin.
