# Supplementary material for: Association between non‐alcoholic fatty liver disease with the susceptibility and outcome of COVID‐19: A retrospective study
Source: J Cell Mol Med. 2021 Nov 10;25(24):11212–20. doi: 10.1111/jcmm.17042 (PMC8650045; doi:10.1111/jcmm.17042)
Supplement: Supplementary file 3 — Table S2 [file JCMM-25-11212-s002.docx]

**Table S2 Multivariate analysis of factors related to severe events of non-elderly COVID-19 patients** **using the COX regression model**

|  | **HR (hazard ratio)** | **95% confidence interval** | **p value** |
| --- | --- | --- | --- |
| **NAFLD** | 1.79 | 0.72, 4.44 | 0.208 |
| **Gender** | 2.52 | 0.93, 6.84 | 0.069 |
| **Hypertension** | 0.25 | 0.09, 0.70 | 0.008 |
| **Cardiovascular disease** | 0.07 | 0.01, 0.58 | 0.013 |
| **Diabetes** | 1.43 | 0.30, 6.83 | 0.983 |
| **Chronic liver disease** | 0.08 | 0.01, 0.76 | **0.028** |

NAFLD, non-alcoholic fatty liver disease.
